# Riding the Digital Wave of Exercise, Health, and Sports Training Optimization

**DOI:** 10.3390/sports12080203

**Published:** 2024-07-25

**Authors:** Rodrigo Zacca, Flávio Antônio de Souza Castro, Rui M. S. Azevedo

**Affiliations:** 1 Research Center in Physical Activity, Health and Leisure (CIAFEL), Faculty of Sports, University of Porto (FADEUP), 4099-002 Porto, Portugal; 2 Laboratory for Integrative and Translational Research in Population Health (ITR), 4050-600 Porto, Portugal; 3 Aquatic Sports Research Group (GPEA), School of Physical Education, Physiotherapy and Dance, Universidade Federal do Rio Grande do Sul, Porto Alegre 90690-200, Brazil; souza.castro@ufrgs.br; 4 Associate Laboratory i4HB—Institute for Health and Bioeconomy, University Institute of Health Sciences—CESPU, 4585-116 Gandra, Portugal; 5 UCIBIO—Applied Molecular Biosciences Unit, Forensics and Biomedical Sciences Research Laboratory, University Institute of Health Sciences (1H-TOXRUN, IUCS-CESPU), 4585-116 Gandra, Portugal

The digital era is opening countless possibilities in “Sport Sciences”; “Public, Environmental, and Occupational Health”; and “Physical Therapy, Sports Therapy, and Rehabilitation” areas [1]. Our Special Issue (Digital Technologies: Applications, Window of Opportunity and Challenges in Exercise, Health and Sports) underlines emerging methodologies, providing some novel solutions for exercise, health, sports, coaches, athletes, and the general population [2,3,4,5]. In fact, digital technologies offer huge potential for development in all these domains. Emerging technologies, such as artificial intelligence, blockchain, and the Internet of Things, are prompting us to rethink approaches to exercise, health, and sports training [1,2,5]. As we embrace these innovations, it is important for the “Sport Sciences”, “Public, Environmental, and Occupational Health”, and “Physical Therapy, Sports Therapy and Rehabilitation” communities to efficiently explore this digital wave. From advanced personalization of exercise and training programs to the use of augmented reality in physical rehabilitation, each innovation opens new gaps to improve fitness, athletic performance, and general health. By navigating this new digital frontier with competence and intelligence, we are not only poised to improve athlete outcomes and the quality of life of the general public, but we are also ready to positively transform the present and future of these areas.

Filtered evidence like systematic reviews and meta-analyses synthesize the literature of RCTs, cohort studies, and, on occasion, case reports. The effort involved demands extensive mental, philosophical, and physical effort from researchers, as well as a monetary investment. Twenty-eight (n = 28) manuscripts were submitted for consideration for our Special Issue. All submitted manuscripts underwent peer review, evaluating the originality and quality of the research, methodological rigor, clarity and relevance of the findings and their applications. Ultimately, the following ten (n = 10) papers were accepted for this Special Issue (one communication, eight articles, and one review):

**Communication**
  (1) Oberhofer, K.; Knopfli, C.; Achermann, B.; Lorenzetti, S.R. Feasibility of Using Laser Imaging Detection and Ranging Technology for Contactless 3D Body Scanning and Anthropometric Assessment of Athletes. *Sports* **2024**, *12*, 92. https://doi.org/10.3390/sports12040092.

**Articles**
  (1) Patti, A.; Giustino, V.; Messina, G.; Figlioli, F.; Cataldi, S.; Poli, L.; Belmonte, G.; Valenza, A.; Amato, A.; Thomas, E.; et al. Effects of Cycling on Spine: A Case–Control Study Using a 3D Scanning Method. *Sports* **2023**, *11*, 227. https://doi.org/10.3390/sports11110227.
  (2) Nagovitsyn, R.S.; Valeeva, R.A.; Latypova, L.A. Artificial Intelligence Program for Predicting Wrestlers’ Sports Performances. *Sports* **2023**, *11*, 196. https://doi.org/10.3390/sports11100196.
  (3) Noteboom, L.; Nijs, A.; Beek, P.J.; van der Helm, F.C.T.; Hoozemans, M.J.M. A Muscle Load Feedback Application for Strength Training: A Proof-of-Concept Study. *Sports* **2023**, *11*, 170. https://doi.org/10.3390/sports11090170.
  (4) Achermann, B.; Oberhofer, K.; Ferguson, S.J.; Lorenzetti, S.R. Velocity-Based Strength Training: The Validity and Personal Monitoring of Barbell Velocity with the Apple Watch. *Sports* **2023**, *11*, 125. https://doi.org/10.3390/sports11070125.
  (5) Cabarkapa, D.V.; Cabarkapa, D.; Philipp, N.M.; Fry, A.C. Impact of the Anatomical Accelerometer Placement on Vertical Jump Performance Characteristics. *Sports* **2023**, *11*, 92. https://doi.org/10.3390/sports11040092.
  (6) Luginbühl, M.; Gross, M.; Lorenzetti, S.; Graf, D.; Bünner, M.J. Identification of Optimal Movement Patterns for Energy Pumping. *Sports* **2023**, *11*, 31. https://doi.org/10.3390/sports11020031.
  (7) Daveri, M.; Fusco, A.; Cortis, C.; Mascherini, G. Effectiveness of Different Modalities of Remote Online Training in Young Healthy Males. *Sports* **2022**, *10*, 170. https://doi.org/10.3390/sports10110170.
  (8) Touloudi, E.; Hassandra, M.; Galanis, E.; Goudas, M.; Theodorakis, Y. Applicability of an Immersive Virtual Reality Exercise Training System for Office Workers during Working Hours. *Sports* **2022**, *10*, 104. https://doi.org/10.3390/sports10070104.

**Review**
  (1) Rossoni, A.; Vecchiato, M.; Brugin, E.; Tranchita, E.; Adami, P.E.; Bartesaghi, M.; Cavarretta, E.; Palermi, S. The eSports Medicine: Pre-Participation Screening and Injuries Management—An Update. *Sports* **2023**, *11*, 34. https://doi.org/10.3390/sports11020034.

These manuscripts were authored by researchers from various countries around the globe, including France, Greece, Italy, Monaco, Poland, Russia, Spain, Switzerland, the Netherlands, and the United States. As editors, we are very grateful for the trust placed in us and are confident that upon reading this Special Issue, you will gain a fresh perspective on “Sport Sciences”; “Public, Environmental, and Occupational Health”; and “Physical Therapy, Sports Therapy, and Rehabilitation”.
